# The association between circadian rhythm of cortisol and shift work regularity among midwives—A multicenter study in Southeast China

**DOI:** 10.3389/fpubh.2022.965872

**Published:** 2022-09-27

**Authors:** Xin-xin Huang, Xiu-min Jiang, Qing-Xiang Zheng, Xiao-Qian Chen

**Affiliations:** Fujian Maternity and Child Health Hospital, College of Clinical Medicine for Obstetrics & Gynecology and Pediatrics, Fujian Medical University, Fuzhou, China

**Keywords:** midwives, cortisol, circadian rhythm, shift regularity, linear mixed-effects models

## Abstract

**Objective:**

This article aims to explore the association between the trends of cortisol rhythm and the regularity of shift work among midwives.

**Methods:**

Midwives from six Southeast Chinese hospitals were recruited through cluster sampling in a multi-center cross-sectional study. Urine samples were collected half an hour after waking up, at 11:00, 19:00, and 23:00 on two consecutive days in a longitudinal cohort. The urinary cortisol was assayed by the chemiluminescence method.

**Results:**

A total of 86 midwives were included in this study, contributing 688 cortisol samples. The midwives displayed a circadian rhythm in cortisol secretion, with zeniths in the morning and nadirs in the evening. The trend of the first day was repeated on the second day. Although the total working hours per week of the two groups, namely the regular shift group (*N* = 43) and the irregular shift group (*N* = 43), were the same, significant main effects of groups (*F* = 62.569, *p* < 0.001), time (*F* = 45.304, *p* < 0.001), and group-by-time interaction (*F* = 226.695, *p* < 0.001) were indicated through linear mixed models. The main effect of day was not statistically significant, with *F* = 0.105 and *p* = 0.746. The fluctuation range of cortisol curve in the group with irregular schedules was slightly lower than that in the group with regular schedules.

**Conclusion:**

Our results may indicate that cortisol was more inhibited in midwives with irregular shift patterns than those with regular shift patterns. It is necessary to further study the relationship between cortisol rhythm and patterns of midwives' shifts in future so as to lay a foundation for hospital managers to develop a more reasonable scheduling system for midwives with the further purpose to minimize their occupational fatigue and ensure the safety of mothers and infants.

## Introduction

Biological rhythm refers to one of the periodic phenomena of life activities, existing in almost all living organisms. In physiological circumstances, when an individual is not exposed to stress, hormonal secretion is regulated by the circadian rhythm. Cortisol, usually assayed from saliva, serum, urine, or hair (1), is a corticosteroid hormone that influences memory consolidation in humans (1) and is a parameter widely accepted by science as the best indicator of stress.

The cortisol release follows a circadian rhythm, which is high in the morning and low at bedtime (2). This circadian rhythm is established between 2 and 9 months after birth (3). This typical secretion pattern is crucial to the functions of all other systems of the human organism (4).

The European Union (EU) defines stress as “a set of neuroendocrine, immunological, and emotional processes and responses” (5) and indicates that stress associated with the workplace is the second most common work problem after musculoskeletal disorders. High and sustained levels of stress can increase the risk of cardiovascular disease, increase susceptibility to infections and mental disorders, and affect the task performance of health professionals (6).

Midwives play an important role in ensuring and promoting the health of pregnant women and newborns (7). Delivery rooms are a place of great pressure, so midwives need to closely observe the labor process, continuously monitor fetal heart, carefully identify critical and abnormal situations, and immediately give effective intervention when necessary. Therefore, their professional skills and abilities for stress are highly required. In recent years, China's universal three-child policy has led to the possibility that some elderly women may give birth to a second or third child. As a result, complications before and during pregnancy have increased, so has the pressure on midwives (8). Shift work is very common in delivery rooms. Some midwives have relatively fixed internals between night shifts or fixed forms of night shifts, while some fluctuate greatly, some attend night shift work in turn, and still some permanent night shift work. They experience fatigue, stress, and burnout due to their workloads. Midwives have reported moderate-to-severe levels of exhaustion on 22–50% of all shift days (9).

There is considerable evidence that acute stressors increase cortisol excretion, for example, a year-long longitudinal study in Germany concluded that waking cortisol was significantly higher among physicians who performed shift work (10).

However, whether irregular shifts increase cortisol disruption is not well understood. In this study, it was hypothesized that irregular shifts could further deepen the effects on cortisol rhythms based on the knowledge that midwives experience changes in stress biomarkers after long-term exposure to stress of daily work. We considered urine cortisol as an indicator to analyze the characteristics of cortisol rhythm of midwives in delivery rooms within 48 h and explore the relationship between cortisol rhythm and scheduling.

## Materials and methods

### Study design and participants

This study was designed as a cross-sectional survey and adhered to STROBE guideline. A descriptive and cross-sectional study was conducted from January 2020 to March 2020 in six Chinese hospitals by means of cluster sampling.

The inclusion criteria were as follows: (1) midwives who were on night shift continuously in 3 months before investigation; (2) midwives who were professionally qualified in labor rooms; (3) midwives who had no mental or physical ailments that could prevent them from their work; (4) midwives who had a regular lifestyle; (5) midwives who had no major life events in 6 months before investigation; (6) midwives who had more than 1 year of midwifery experience in labor rooms; (7) midwives who had not smoked, drunk coffee or tea 12 h before urine samples were collected; (8) midwives who participated in this study voluntarily.

The exclusion criteria were as follows: (1) midwives who were absent from work due to illness for more than 15 days in the last 3 months; (2) midwives who smoked or had ever smoked for more than 5 years; (3) midwives who had an abuse of alcoholic beverages or had ever had such a history for more than 5 years; (4) midwives who were using medications that influence the hypothalamic–pituitary–adrenal (HPA) axis (for example glucocorticoids, steroids, beta-blockers, antidepressants, melatonin, or any other psychoactive drugs); (5) midwives who were medically diagnosed with neurological or psychiatric illness; (6) midwives who had such health-related problems as diabetes, high blood pressure, or cancer; (7) midwives who were in menstrual or ovulation period, pregnancy, or lactation; (8) midwives who were diagnosed with acute diseases or in an acute attack stage of chronic diseases.

### Outcome measurements

After the distribution of urine packs, midwives were informed to collect their urine samples at eight time points. The subjects collected their urine according to the specific time intervals they were told to follow. Urine samples were collected on two consecutive working days, four times a day, namely, half an hour after waking up, at 11:00, 19:00, and 23:00. Thus, urine samples were obtained from each subject at four intervals within two consecutive days, which were classified as T_1_, T_2_, T_3_, and T_4_ on the first day and T_1_, T_2_, T_3_, and T_4_ on the second day.

About 2 mls of all urine samples was taken and centrifugated at 3000 rotational speed per minute (rpm) for 8 min and then was stored in a refrigerator at −80°C for further testing. After that, each portion of centrifugate was tested for the level of cortisol by ELISA kits (The brand of Access Cortisol: Beckman Coulter, Catalog number: 33600. Testing instrument: Access) and chemiluminescence method.

### Data collection

Urine samples and data were collected from midwives working in the six hospitals involved in this study. Informed consent was obtained from the nursing departments of these hospitals prior to the investigation. A project work group was established to develop a unified survey and questionnaire to carry out the investigation through unified methods. The researchers trained six charge nurses in the maternity wards of the sample hospitals and presented the study protocols. After unified training and assessment, the charge nurses then explained in detail the objective of the study, questionnaire filling method, and the method of urine specimen collection to the subjects who met the inclusion criteria. The subjects were asked to wrap their urine samples in a separate black plastic bag and store them temporarily in a refrigerator at 4°C. Researchers visited each sample hospital once or twice a day and sent urine samples collected in ice packs to laboratories for unified testing until all urine samples were collected and tested.

A questionnaire was conducted 1–3 days before the urine test. In the baseline questionnaire, each midwife was asked “Is your night shift regular?” Irregular shifts were defined as the intervals between night shifts or the forms of night shift (e.g., night shift before mid-night, night shift after mid-night, and overnight shift) which were not fixed in the month prior to the survey. Otherwise, it was defined as a regular one. The head nurses classified participants based on their schedules for the month preceding the survey. Participation was voluntary and anonymous. The head nurses assisted in distributing the questionnaires and reminded the midwives to complete them. The questionnaires were sealed for confidentiality and given to the head nurses when completed.

### Data analysis

Statistical analysis was performed by SPSS statistical software (version 26.0, SPSS Inc., Chicago, IL, USA). Data that conformed to the normal distribution were expressed as mean ± standard deviation, and the differences between groups were compared through the independent-samples test. For quantitative data that did not conform to the normal distribution, the median (interquartile range) was used, and the differences between groups were compared through the Wilcoxon rank sum test. The categorical variables were presented as numbers (n) and percentages (%), and the differences between groups were compared with the chi-square test. Fisher's exact test was performed for categorical variables with small, expected numbers. The distributions of the cortisol levels were skewed to the right, so we transformed the data into logarithm with its base of 10. The log-transformed data were then normally distributed. Linear mixed-effects models (LMMs) were used to test changes in outcomes over time, and age was used as a moderating factor. A two-tailed significance test was applied for all comparisons, and *P* = < 0.05 indicated statistical significance.

## Results

### General demographic

Of the 100 participants, nine did not attend the night shifts and five were excluded due to incomplete data. The actual samples (*n* = 86) were composed of 50% regular night shift group (*n* = 43) and 50% (*n* = 43) irregular night shift group. We surveyed the subjects' ages, education, marital status, and so on. Table 1 shows the characteristics of the study samples: A total of 86 midwives were with a median age of 29 (26, 34) years, averaging almost 39 (35, 47.13) h of work per week, and 4 (2, 5.25) night shift days per month. Regarding health-related behaviors, no midwives smoked or had risky alcohol use. The majority (69.77%) were engaged in regular physical activity, and the majority (77.9%) had normal body mass index. There was no statistical difference in basic characteristics and lifestyle between the two groups.

**Table 1:** Demographic data of study participants.

**Variation**		**Regular shift group (*n* = 43)**	**Irregular shift group (*n* = 43)**	**Total**	**z/χ^2^/t**	***p***
Age		29(26,36)	29(26,32)	29(26,34)	0.757	0.449
Marital status	Unmarried	17	16	33	0.049	0.825
	Married	26	27	53		
Level of education	Junior college	18	21	39	0.422	0.516
	Bachelor's degree or above	25	22	47		
Years of working in delivery room	≤ 10 year	31	33	64	—*	0.078
	11–20 year	7	10	17		
	≥21 year	5	0	5		
Working hours per week (hour)		40(35,47)	39(35,48)	39(35,47)	0.636	0.525
Night shift days per month (day)		4(2,6)	4(2,5)	4(2,5.25)	0.226	0.821
Body mass index (BMI)		21.56 ± 2.54	20.35 ± 2.38	20.80 ± 2.49	1.714	0.090
Physical activity	Inactive	13	13	26	0.003	1.000
	Active	30	30	63		
The main forms of night shift	16:00–24:00	11	10	21	—*	0.694
	0:00–8:00	16	12	28		
	16:00–8:00 at next day	12	17	29		
	8:00–16:00 and 0:00–8:00 at next day	4	4	8		

^*^Fisher's exact test.

### Cortisol rhythm

**Figure 1:**
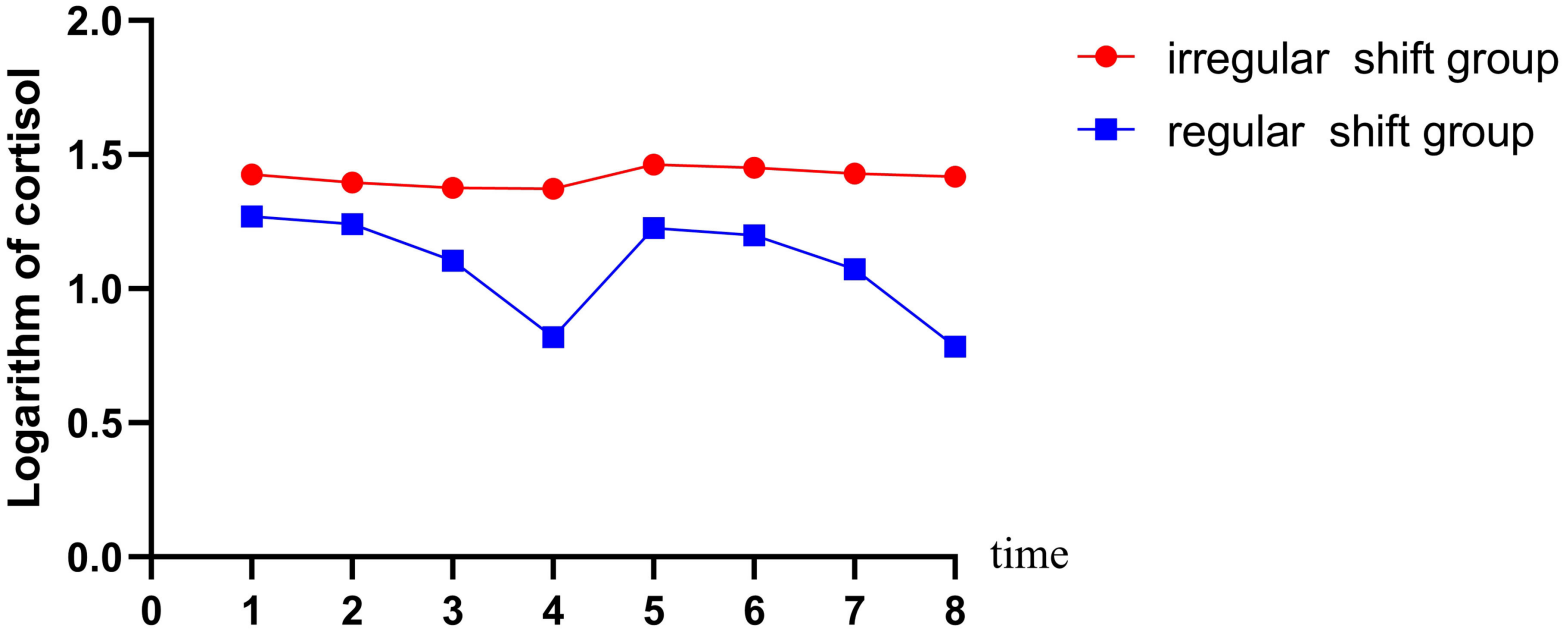
Diurnal pattern of cortisol secretion according to shift work at baseline. 1 = 30 min after waking on the first day; 2 = 11:00 on the first day; 3 = 19:00 on the first day; 4 = 23:00 on the first day; 5 = 30 min after waking on the second day; 6 = 11:00 on the second day; 7 = 19:00 on the second day; 8 = 23:00 on the second day. The blue line represents cortisol pattern for midwives with regular shifts (*N* = 43). The red line represents cortisol pattern for midwives with irregular shifts (*N* = 43).

Raw cortisol data are shown in Table 2, and the logarithmic distribution of the cortisol of the midwives in the two groups for two consecutive days is shown in Figure 1, suggesting a significant change of cortisol release over time.

**Table 2:** Cortisol levels between the two groups [P_50_(P_25_, P_75_)].

	**Regular shift group (*n* = 43)**	**Irregular shift group (*n* = 43)**
T_1_ on the first day	19.05 (14.13,28.84)	25.7 (16.6,45.71)
T_2_ on the first day	17.78 (13.18,25.7)	22.91 (16.98,39.81)
T_3_ on the first day	14.45 (9.12,17.78)	23.99 (15.49,34.67)
T_4_ on the first day	6.46 (3.02,12.88)	21.88 (14.45,38.02)
T_1_ on the second day	16.6 (12.02,24.55)	28.18 (18.2,50.12)
T_2_ on the second day	16.6 (10.72,27.54)	25.12 (19.5,45.71)
T_3_ on the second day	12.3 (9.12,16.22)	26.3 (17.78,44.67)
T_4_ on the second day	5.5 (3.72,12.3)	28.84 (16.98,40.74)

The average logarithm of day was the highest half an hour after waking up and then declined gradually over the day, reaching their lowest point at 23:00. Half an hour after waking up the next day, the cortisol level was close to that of the first day, and the changing trend of the first day was repeated. Midwives with regular shifts had a greater range of cortisol changes. The *p*-values for the changes in time, group, day, and group^*^time were calculated by linear mixed model. The analysis considered time, group, day, and group^*^time as fixed effects, subjects' numbers as random effects, and their ages as a covariate.

A design of 2 (regular night shift/irregular night shift) × 4 (four duration points) was carried out to assess the differences of the two groups across time. We found significantly lower cortisol levels in regular shift midwives than those in irregular shift midwives (*F* = 62.569, *p* < 0.001). The changes of cortisol levels of midwives over time were obvious, with *F* = 45.304 and *p* < 0.001. The group-by-time interaction was also obvious, with *F* = 226.695 and *p* < 0.001. The main effect of day was not statistically significant, with *F* = 0. 105 and *p* = 0.746. The estimates of fixed effects are shown in Table 3.

**Table 3:** Estimates of fixed effects.

**Parameter**	**Estimate (95% CI)**	***p***
Intercept	0.85(0.62,1.09)	
[group = 1]	0.60(0.49,0.71)	< 0.001
[time = 1]	0.45(0.39,0.52)	< 0.001
[time = 2]	0.42(0.36,0.49)	< 0.001
[time = 3]	0.28(0.22,0.35)	< 0.001
[day = 1]	0(− 0.03,0.02)	0.746
Age	0(− 0.01,0.01)	0.629
[time = 1] * [group = 1]	−0.41(−0.5, −0.32)	< 0.001
[time = 2] * [group = 1]	−0.4(−0.49, −0.31)	< 0.001
[time = 3] * [group = 1]	−0.27(−0.36, −0.18)	< 0.001

*Group = 1: irregular shift group.

Time = 1: 30 min after waking.

Day = 1: the first day.

### Main effect derived from the comparison between the groups

The main effect from the comparison between the two groups was that the mean logarithmic cortisol of the regular group was 0.33 (0.25–0.41) lower than that of the irregular group, which was statistically significant (*P* < 0.001).

### Main effect derived from the comparison of eight time points

Further analysis through pairwise comparison was carried out to test whether the differences between the adjacent time points were significant, as shown in Table 4. The logarithm of cortisol began to decline (*p* < 0.001) at 19:00, and it dropped to its lowest level (*p* < 0.001) at 23:00.

**Table 4:** Pairwise comparisons of time effect.

	**Adjusted difference** **(95% CI)**	***P***
T_1_ vs. T_2_	0.03(−0.01,0.06)*	0.155
T_2_ vs. T_3_	0.08(0.04,0.11)*	< 0.001
T_3_ vs. T_4_	0.15(0.1,0.19)*	< 0.001

*Linear mixed-effects model adjusted for age, day, and subject as a random effect.

T_1_ = 30 min after waking; T2 = 11:00; T_3_ = 19:00; T_4_ = 23:00.

## Discussion

In this study, the aim was to examine the impact of shift work regularity on circadian rhythm of cortisol. We used cortisol and its variations to reflect the daily stress experienced by midwives in hospital delivery rooms. The midwives' urinary cortisol levels varied based on different shift schedules for the two groups though their overall working hours were the same and their night shift days were approximate. Linear mixed models found that time, group, and time^*^group were statistically significant.

Further comparison showed that the cortisol of midwives began to decrease gradually half an hour after waking up in the morning, till to the lowest point at 23:00 on the first day. Compared with that lowest point, it rose obviously 30 min after waking up in the next day, and the trend of the first day was repeated until another trough appeared at 23:00. The fluctuation range of cortisol curve was slightly lower in the group of midwives with irregular work schedules. Our data confirmed that the variations of cortisol of midwives in the two groups were different, indicating that irregular schedules exerted more impact on the circadian rhythms than regular schedules among midwives on shift.

According to the latest research findings, cortisol, as a health biomarker of circadian rhythm, has been extensively investigated (11).

The cortisol release is typically in line with circadian rhythm, which is believed to grow within the first hour after waking up and reach its pinnacle around 30–45 min after waking up. It gradually dwindles during the day and falls to the minimum at mid-night (11, 12). Such circadian rhythm was verified through the logarithmic distribution of the 86 midwives' cortisol for two consecutive days in our study.

It is well known that both acute and chronic stress imply changes in cortisol. The circadian disruption induced by shift work would impair the functioning of the hypothalamic–pituitary–adrenal (HPA) axis which regulates the biological response to stressful stimuli (1). Immediate release of cortisol is an adaptive response to acute stress, but repeated or long-lasting stress may have negative effects on human bodies, that is, failure to exhibit an expected biomarker's circadian cycle or to recover after a stressor (13). Saliva cortisol level flat mirror is the foundation of HPA axis dysfunction (14).

Our study found that midwives with irregular shifts had relatively high cortisol levels at all the four points of the day (including waking up in the morning and going to bed in the evening), and their cortisol tendency from the morning to the evening was relatively stable compared with that of midwives with regular shits. Since the cortisol response was positively related to work stress, it was further confirmed that they had problems of great pressure and easy fatigue in work.

As a special medical group, midwives shoulder the double pressure of ensuring the health of delivery women and newborns. They often must deal with various special situations related to predelivery, delivery, and postpartum and are faced with many occupational risks, such as potential unsafe factors during labor, sharp instrument injury, and maternal and infant blood and body fluid exposure. The work of midwives demands constant attention, focused memory, and decision-making, and they often need to make quick decisions in emergency. For example, whether the fetus has intrauterine asphyxia, whether an emergency C-section is needed. Does the maternal labor progress stagnate? Whether the delivery women may face such situations as amniotic fluid embolism, postpartum hemorrhage, diffuse intravascular hemorrhage, and other life-threatening emergency. Individuals who work overnight or in shifts often experience circadian disruption and sleep restriction. The previous assessment of the psychological behavior scale of midwives in China found that the job burnout of midwives was at a high level (15).

It is reported that the stress of nursing teams has been evaluated worldwide with pressure scales (4). Since the scale assessment is highly subjective, we speculate that objective laboratory indicators can be referred as biomarkers of midwives' stress intensity in future, but presently, there are few studies on markers of stress in midwives. It has been found that the morning salivary cortisol concentration of emergency nurses was well correlated with the modified Mental Health Professional Stress Scale (PSS); therefore, the morning salivary cortisol sample can be applied to reflect self-perceived stress (16).

In the past, some epidemiological studies on shift work and cortisol have produced inconsistent results. For example, it was reported during shift work in emergency rooms, the salivary cortisol levels were increased (17). Sanaa found that the experimental group of nurses with night shifts had significantly higher levels of cortisol than the control group of nurses with day shifts only (18).

A similar result was found by Muhammad (19). These above-mentioned studies supported the idea that shift work can alter the cortisol patterns. However, a Canadian study among paramedics did not find any relationship between shift work and cortisol secretion (20). Those studies involved medical personnel outside obstetrics. We consider that the conclusion may be related to the nature, working environment, and working content of the specific night shift, which needs to be further analyzed in a unified and standardized way.

The sampling methods and sampling timing may affect the detection results, for example, the serum cortisol sampling time conducted in a hospital is relatively fixed, resulting in a “cortisol awakening response” (21) which is conceptualized as “a sharp increase in cortisol levels across the first 30–45 min following morning awakening” and cannot be accurately captured. In our study, we designed a urine sample of cortisol that can be easily sampled and administered by midwives at home. The first sample of the day was taken 30 min after waking up in the morning, just at the theoretical peak of cortisol secretion, so there was no specific time for collection. On the contrary, this is the time when most midwives are ready to leave home for work, which means they are about to be counted as working status and are psychologically ready to face the stress of the day.

On the other hand, those studies only reported whether they were engaged into shift work and aimed at medical personnel outside the obstetrics. We believe that the conclusion of the correlation between cortisol and shift work may be related to the nature, environment, and content of the specific night shift, which needs further analysis. The nature and pressure of midwives' work are different from those of nurses, so it is necessary to study separately the pressure status of midwives and to analyze accurately their stress index and help midwifery managers to carry out accordingly manpower management and occupational fatigue protection. There were few previous studies on the variation trend of midwives' shifts and cortisol levels, so the conclusion of our study can provide innovative reference for the management of the allocations of midwife human resources and the obstetrical midwifery technical forces. Given the nature of natural childbirth, it is not possible to completely avoid shift work for midwives, and it is necessary to reasonably adjust the number of shifts to minimize disruption to their circadian rhythms. Previous large sample data from the research hospital showed that the number of natural newborns during the day was nearly the same with that during the night (22). This study found that even working for the same hours with midwives with regular shifts each week, midwives with irregular shifts had higher cortisol level, which indicated that their work pressure was higher. This may be an area requiring further research and improvement in future.

Cumulative epidemiological evidence suggested that shift work could exert harmful effects on human health. Several chronic health conditions have been identified related to it (23).

According to a handful studies with respect to recovery of cortisol circadian rhythm after shift work, it would be more desirable to allow sufficient time for rest between shifts (24). The time from shift work to full recovery can cover at least 2 days in the case of frequent interruption of normal circadian rhythms, and even 3–4 days if they were severely disrupted (25). Midwives in the United States whose work shifts are longer than 12 h have higher rates of excessive daytime sleepiness than midwives whose work shifts are not longer than 12 h (26). Structural changes for midwives to ensure personal and patients' safety include changing the workflow, environment, or institutional policies. This study found no statistical significance of the main effect of the number of days, considering that it was related to the small number of days studied, but it is also possible that the regularity of the shift only affects the level of cortisol change within 24 h. Therefore, it is recommended to extend the days for study in future to explore the characteristics of cortisol changes within several days after the regular/irregular group midwives just complete the night shifts and to determine which shift cycle and shift form of midwives are more desirable for their circadian rhythm. It is also suggested that a relatively stable interval between shifts be maintained to help the midwives to restore their circadian rhythms after work at night and get sufficient rest at intervals of work.

## Conclusion

From the research, it is clear that irregular shifts can be stressful to many midwives. The evidence from available research on shift work and cortisol circadian rhythm would provide meaningful information to future interventions regarding work schedule management. It was reported that the cortisol status may influence overall health condition as well as such indispensable work skills as attention (27). Midwives need to be very focused to observe labor process and handle it decisively in their jobs. Therefore, to improve the quality of their work, to protect their physical and mental health, and to ensure the safety of mothers and infants, hospitals need to intervene in their managements to develop a physiologically more appropriate and adaptive shift schedule to fully restore midwives' circadian rhythm and prevent possible disruption after their night work. In the meantime, accurate, valid, and non-invasive methods need to be applied to understand and monitor the midwives' levels of work stress. According to the test results, early warning intervention may be given. This study provides a new basis for protecting midwives' physical and mental health and the safety of mothers and infants.

### Strengths

This was a multi-center study on six Chinese hospitals, which was representative to some extent. Urine cortisol was collected and measured at eight time points for two consecutive days to obtain reliable trends of its variation. To the authors' knowledge, the relationship between shift regularity and cortisol rhythm had been rarely reported in the literature, so this study provided an innovative basis for a more rational dispatch of midwives.

### Limitations

First, the influence of additional stress sources outside the workplace was not evaluated. Second, we did not record in detail the accurate amount of job of each participant during their shifts.

## Data availability statement

The raw data supporting the conclusions of this article will be made available by the authors, without undue reservation.

## Ethics statement

The studies involving human participants were reviewed and approved by Human Subjects Ethics Committee of Fujian Provincial Maternity and Children's Hospital. The patients/participants provided their written informed consent to participate in this study.

## Author contributions

X-mJ: study design, quality control, and manuscript revision. X-xH: data collection, data analysis, manuscript drafting, and manuscript revision. Q-XZ and X-QC: data interpretation and data collection. All authors contributed to the article and approved the submitted version.

## Funding

This study was supported by a grant from Social Development Guiding Project Fund from Fujian Science and Technology Department (No. 2019Y0059).

## Conflict of interest

The authors declare that the research was conducted in the absence of any commercial or financial relationships that could be construed as a potential conflict of interest.

## Publisher's note

All claims expressed in this article are solely those of the authors and do not necessarily represent those of their affiliated organizations, or those of the publisher, the editors and the reviewers. Any product that may be evaluated in this article, or claim that may be made by its manufacturer, is not guaranteed or endorsed by the publisher.

## References

[1] Miller GE Chen E Zhou ES. If it goes up, must it come down? Chronic stress and the hypothalamic-pituitary-adrenocortical axis in humans. Psychol Bull. (2007) 133:25–45. 10.1037/0033-2909.133.1.25 17201569

[2] Gallagher TF Yoshida K Roffwarg HD Fukoshima DK Weitzman ED Hellman L. ACTH and cortisol secretory patterns in man. J Clin Endocrinol Metab. (1973) 36:1058–68. 10.1210/jcem-36-6-1058 4350348

[3] Donzella B Kertes DA Gunnar MR. Developmental changes in baseline cortisol activity in early childhood: relations with napping and effortful control. Dev Psychbiol. (2004) 45:125–33. 10.1002/dev.20026 15505801

[4] Cordova A. Fisiología Dinamica. Masson-Elsevier: Madrid, Spain (2003).

[5] Pérez-Valdecantos D Caballero-García A Del Castillo-Sanz T Bello HJ Roche E Córdova A. Stress salivary biomarkers variation during the workday in emergencies in healthcare professionals. Int. J. Environ. Res. Public Health. (2021) 18:3937. 10.3390/ijerph18083937 33918537 PMC8070075

[6] Cozma S Dima-Cozma LC Ghiciuc CM Pasquali V Saponaro A Patacchioli FR. Salivary cortisol and -amylase: subclinical indicators of stress as cardiometabolic risk. Braz J Med Biol Res. (2017) 50:e5577. 10.1590/1414-431x20165577 28177057 PMC5390531

[7] Beal MW Batzli ME Hoyt A. Regulation of certified nurse-midwife scope of practice: change in the professional practice index, 2000 to 2015. J Midwifery Womens Health. (2015) 60:510–8. 10.1111/jmwh.12362 26382028

[8] Li C Wang H Wu Y Duan J. Stress cardiomyopathy should be considered with unexpected hypotension in pregnant women. Clin Case Rep. (2020) 8:1265–1268. 10.1002/ccr3.2758 32695372 PMC7364092

[9] Tremaine R Dorrian J Paterson J. Actigraph estimates of the sleep of Australian midwives: the impact of shift work. Biol Res Nurs. (2013) 15:191–9. 10.1177/1099800411422249 21998448

[10] Li J Bidlingmaier M Petru R Pedrosa Gil F Loerbroks A Angerer P. Impact of shift work on the diurnal cortisol rhythm: a one-year longitudinal study in junior physicians. J Occup Med Toxicol. (2018) 13:23. 10.1186/s12995-018-0204-y 30123312 PMC6090626

[11] Zare S Hemmatjo R ElahiShirvan H Malekabad AJ Kazemi R Nadri F. Weighing and modelling factors influencing serum cortisol and melatonin concentration among workers that are exposed to various sound pressure levels using neural network algorithm: an empirical study. Heliyon. (2020) 6:e05044. 10.1016/j.heliyon.2020.e05044 33033770 PMC7534182

[12] Czeisler CA Klerman EB. Circadian and sleep-dependent regulation of hormone release in humans. Recent Prog Horm Res. (1999) 54:97–130. 10548874

[13] Matthews K Schwartz J Cohen S. T. Seeman Diurnal cortisol decline is related to coronary calcification: CARDIA study. Psychosom Med. (2006) 68:657–61. 10.1097/01.psy.0000244071.42939.0e 17012518

[14] Nater UM Hoppmann CA Scott SB. Diurnal profiles of salivary cortisol and alpha amylase change across the adult lifespan: evidence from repeated daily life assessments. Psychoneuroendocrinology. (2013) 38:3167–71. 10.1016/j.psyneuen.2013.09.008 24099860 PMC3844069

[15] Kaina F. Research on the Relationship among Midwives' Job Burnout, Resilience and Coping Style. Master's thesis of Nanchang University.

[16] Yang Y Koh D Ng V Chia SE. Salivary cortisol levels and work-related stress among emergency department nurses. J Occup Environ Med. (2001) 43:1011–8. 10.1097/00043764-200112000-00003 11765672

[17] Kent J Thornton M Allan F Erin H Fitzgibbons S Sava J. Acute provider stress in high stakes medical care: Implications for trauma surgeons. J Trauma Acute Care Surg. (2020) 88:440–5. 10.1097/TA.0000000000002565 32107357

[18] El-Benhawy SA El-Tahan RA Nakhla SF. Exposure to radiation during work shifts and working at night act as occupational stressors alter redox and inflflammatory markers. Arch Med Res. (2021) 52:76–83. 10.1016/j.arcmed.2020.10.001 33039210

[19] Muhammad DH Qadir FA. “Effects of Night Shift Working on Some Immunological, Prostate specific antigen, Cortisol level and Malon-dialdehyde in ale Nurses at Hawler City,” in AIP Conference Proceedings. (2017). 10.1063/1.5004312

[20] Wong IS Ostry AS Demers PA Davies HW. Job strain and shift work influences on biomarkers and subclinical heart disease indicators: a pilot study. J Occup Environ Hyg. (2012) 9:467–77. 10.1080/15459624.2012.693831 22708722

[21] Stalder T Kirschbaum C Kudielka BM Adam EK Pruessner JC Wüst S. Assessment of the cortisol awakening response: expert consensus guidelines. Psychoneuroendocrinology. (2016) 63:414–32. 10.1016/j.psyneuen.2015.10.010 26563991

[22] Xinxin H Xiumin J Xiuwu L. A study on the birth time trend of babies delivered vaginally. Maternal Child Health Care China. (2016) 31:3597–99.10750409

[23] Kecklund G Axelsson J. Health consequences of shift work and insufficient sleep. BMJ. (2016) 355:i5210. 10.1136/bmj.i5210 27803010

[24] Jensen MA Hansen ÅM Kristiansen J Nabe-Nielsen K Garde AH. Changes in the diurnal rhythms of cortisol, melatonin, and testosterone after 2, 4, and 7 consecutive night shifts in male police officers. Chronobiol Int. (2016) 33:1280–92. 10.1080/07420528.2016.1212869 27715321

[25] Åkerstedt T Kecklund G Gillberg M Lowden A Axelsson J. Sleepiness and days of recovery. Transp Res Part Trac Psychol Behav. (2000) 3:251–61. 10.1016/S1369-8478(01)00009-2

[26] Arbour MW Gordon IK Saftner M Tanner T. The experience of sleep deprivation for midwives practicing in the United States. MIDWIFERY. (2020) 89:102782. 10.1016/j.midw.2020.102782 32554134

[27] Bortolotto I de Brum AP Guecheva TN de Souza LM de Paula-Ramos AL Trindade C. DNA damage, salivary cortisol levels, and cognitive parameters in a nursing team. Mutat Res. (2021) 861–862:503300. 10.1016/j.mrgentox.2020.503300 33551101

